# PROFILE AND LEARNING CURVE OF BRAZILIAN SURGEONS REGARDING SHOULDER ARTHROSCOPY

**DOI:** 10.1590/1413-785220253302e288584

**Published:** 2025-10-13

**Authors:** GUILHERME VIEIRA LIMA, PAULO SANTORO BELANGERO, RENATO AROCA ZAN, RAFAEL FUCHS LAZARINI, BERNARDO BARCELLOS TERRA, MARCELO CARVALHO KRAUSE GONCALVES, MARIA ISABEL POZZI GUERRA, LUCAS BRAGA JACQUES GONÇALVES, MARCIO DIEGO CASTRO TEIXEIRA, SANDRO DA SILVA REGINALDO, LUIS ALFREDO GOMEZ-VIEIRA

**Affiliations:** 1. Sociedade Brasileira de Cirurgia de Ombro e Cotovelo (SBCOC), Sao Paulo, SP, Brazil.; 2. Faculdade de Medicina do ABC, Disciplina de Ortopedia e Traumatologia, Grupo de Ombro e Cotovelo, Santo Andre, Sao Paulo, SP, Brazil.; 3. Universidade Federal de Sao Paulo (UNIFESP), Escola Paulista de Medicina Disciplina de Medicina Esportiva, Departamento de Ortopedia e Traumatologia, Sao Paulo, SP, Brazil.; 4. Universidade Federal de Sao Paulo (UNIFESP), Departamento de Cirurgia da Mao e Membro Superior, Sao Paulo, SP, Brazil.; 5. Hospital Felicio Rocho Biocor, Rede D’Or, Belo Horizonte, Minas Gerais, MG, Brazil.; 6. Santa Casa de Vitoria, Vitoria, Espirito Santo, ES, Brazil.; 7. Instituto de Traumatologia e Ortopedia Romeu Krause, Recife, Pernambuco, PE, Brazil.; 8. Hospital Madre Teresa, Servico de Ortopedia, Belo Horizonte, Minas Gerais, MG, Brazil.; 9. Universidade Federal de Goias, Hospital das Clinicas/EBSERH, Unidade de Traumatologia e Ortopedia, Goiania, Goias, GO, Brazil.; 10. Hospitais Portugues e MaterDei, Servico de Cirurgia de Ombro e Cotovelo, Salvador, Bahia, BA, Brazil.

**Keywords:** Arthroscopy, Shoulder, Learning, Education, Artroscopia, Ombro, Aprendizado, Educação

## Abstract

**Objective:**

The aim of this study is to investigate the number of surgeries necessary for an orthopedic surgeon specialized in shoulder surgery to become proficient in performing arthroscopy. This is an original article with level V evidence.

**Methods:**

A cross-sectional study was designed to examine the perspectives of surgeons at various stages of their careers, using an online questionnaire.

**Results:**

A total of 251 participants responded. The most prevalent training period was more than 15 years of experience. The proficient level was the most prevalent. In evaluating the number of arthroscopies by proficiency level, all agreed that for the specialist level, a total of over 500 arthroscopies is necessary. Most respondents judged 31 to 50 arthroscopies as necessary to perform safely. According to the methodologies, the best-rated were acting as the lead surgeon and training on cadavers.

**Conclusion:**

The study showed that 31 to 50 cases are necessary to perform shoulder arthroscopy safely, and over 500 cases to reach the specialist level. Participation as the lead surgeon and training on cadavers were rated as very important in specialist training. Evidence Level V; Expert Opinion.

## RESUMO

**Objetivo:**

investigar o número de cirurgias necessárias para que um ortopedista com especialização em cirurgia de ombro se torne seguro na realização de uma artroscopia. Trata-se de um artigo original com nível de evidência V.

**Métodos:**

Realizado um estudo transversal projetado para examinar as perspectivas dos cirurgiões em vários estágios de sua carreira, utilizando um questionário online.

**Resultados:**

Obtivemos 251 participantes. O tempo de formação mais prevalente foi mais 15 anos de experiência. O nível proficiente foi o mais prevalente e o número de artroscopias anuais mais prevalente foi a faixa de 51 a 99 cirurgias. Em relação ao nível de aptidão, para o nível especialista é necessário um número total de artroscopias superior a 500. Para realizar com segurança a maioria dos entrevistados julgam necessários de 31 a 50 artroscopias. De acordo com as metodologias as melhores avaliadas foram: atuação como cirurgião principal e treinamento em cadáver.

**Conclusão:**

O estudo mostrou que para realizar uma artroscopia de ombro com segurança são necessários 31 a 50 casos e mais de 500 casos para alcançar o nível de especialista. Participação como cirurgião principal e treinamento em cadáveres foram avaliados como muito importantes na formação do especialista. Nível de Evidência V; Opinião de Especialista.

## INTRODUCTION

Shoulder arthroscopy is a well-established procedure offering various advantages such as less trauma to soft tissues, better evaluation of the glenohumeral joint, subacromial space, and rotator cuff, lower infection rates, less postoperative pain, and earlier return to physical and work activities.^1,2^ This surgical modality is widely used in the treatment of rotator cuff injuries, joint instability, certain types of fractures, among other pathologies.^3-5^

Although the surgical learning curve has been investigated for procedures such as hip and elbow arthroscopy,^6-8^ there is a lack of literature on shoulder arthroscopy. How many surgeries are necessary for a surgeon to become a specialist in performing shoulder arthroscopy? Is there a difference in the perception of this number between more experienced surgeons and newly graduated ones? What are the best learning tools for shoulder arthroscopy? These questions remain unanswered.

The main objective of this study is to investigate the number of surgeries necessary for an orthopedic surgeon specializing in shoulder and elbow surgery in Brazil to become proficient and a specialist in performing shoulder arthroscopy. Additionally, we aim to compare the perceptions of more experienced surgeons and newly graduated ones. Secondly, we aim to evaluate the best training tools for shoulder arthroscopy.

The probable hypothesis is that there will be significant perspective differences between trainees and more experienced surgeons regarding the number of cases necessary to achieve each skill level, but the most valuable training tool will be similar regardless of the surgeon’s level.

## MATERIAL AND METHODS

This is a cross-sectional study designed to examine the current perspectives of orthopedic surgeons at various career stages using an online questionnaire for data collection.

We included orthopedic doctors with a specialty title in Orthopedics and Traumatology (TEOT) by the Brazilian Society of Orthopedics and Traumatology (SBOT) practicing in Brazil, who have specialized training in shoulder and elbow surgery and are registered in the Brazilian Society of Shoulder and Elbow Surgery database. Those who did not agree with the informed consent form or had incomplete or unfilled forms were excluded.

The questionnaire was emailed to all addresses registered in the SBCOC database in 2022. The document sent included a summary of the research and a link directing the participant to the questionnaire on the Google Forms platform as per Appendix 1.

Participants were subdivided into different groups for comparative analyses based on the following factors: years of training in shoulder surgery (≤5, 6-10, 11-14, and ≥15 years), whether they were a training program coordinator in shoulder surgery, the number of shoulder arthroscopies performed annually (≤10, 11-20, 21-30, 31-50, 51-99, and ≥100), self-assigned skill level (initial, safe, competent, proficient, or specialist).

We sought among Brazilian surgeons to (1) compare the perspectives of experienced surgeons and newly graduated trainees; (2) compare these recommended case volume requirements based on physician demographics, training, professional experience, and skill level; and (3) determine which training methodologies participants and trainees considered most valuable in acquiring shoulder arthroscopy skills.

The study was approved by the ethics committee CAAE: 61507822.5.00000.5125 according to the requirements of the Declaration of Helsinki. Opinion Number: 5.624.585.

### Statistical Analysis

Participants’ demographic data were used descriptively, including means with standard deviation, percentages, and variance, where appropriate. After evaluating the data for any parametric or non-parametric assumptions, all continuous variables were compared between the previously mentioned groups using the chi-square test. When comparing means between groups, the mean difference and 95% confidence interval (CI) were determined. P-values <0.05 were considered significant. Statistical analysis was performed using SPSS V20, Minitab 16, and Excel Office 2010 software.

## RESULTS

The questionnaire was emailed to 934 individuals according to the SBCOC database. Forty-four email addresses were incorrectly registered, and 639 individuals did not respond to the questionnaire. We included 251 participants with a mean age of 43.2 ± 1.2 years (Table 1), ranging from 25 to 72 years, with 96.8% being male.

**Table 1:** Full Descriptive Analysis of Age.

Age	
Mean	43.2
Median	42
Standard Deviation	9.7
CV	22%
Q1	36
Q3	47.5
Min	25
Max	72
N	251
CI	1.2

The most prevalent training period was ≥15 years, with 35.1%, but this is not statistically different from the 27.9% with ≤5 years (p= 0.084).

The proficient level in shoulder arthroscopy was the most prevalent with 55.8% of doctors, and the most prevalent number of annual arthroscopies was in the range of 51 to 99 surgeries at 29.5%.

The southeast region was the most prevalent geographical area of practice at 53.4% (Table 2).

**Table 2:** Distribution of Qualitative Factors.

		N	%	P-value
Leads a training group	No	157	62.5%	<0.001
	Yes	94	37.5%	
Skill level in shoulder arthroscopy	Proficient	140	55.8%	Ref.
	Specialist	53	21.1%	<0.001
	Competent	46	18.3%	<0.001
	Secure	9	3.6%	<0.001
	Beginner	3	1.2%	<0.001
Number of shoulder arthroscopies performed per year	≤10	15	6.0%	<0.001
	11-20	26	10.4%	<0.001
	20-30	1	0.4%	<0.001
	21-30	28	11.2%	<0.001
	31-50	35	13.9%	<0.001
	51-99	74	29.5%	Ref.
	≥100	72	28.7%	0.844
Holds SBCOC certification	No	16	6.4%	<0.001
	Yes	235	93.6%	
Region	Southeast	134	53.4%	Ref.
	Northeast	43	17.1%	<0.001
	South	36	14.3%	<0.001
	Central-West	29	11.6%	<0.001
	North	9	3.6%	<0.001
Sex	Female	8	3.2%	<0.001
	Male	243	96.8%	
Years of experience	Residency (2022)	4	1.6%	<0.001
	≤5 years	70	27.9%	0.084
	6- 10 years	49	19.5%	<0.001
	11-14 years	40	15.9%	<0.001
	≥15 years	88	35.1%	Ref.

Regarding training time and mean age of doctors, there was statistical significance; the mean age for professionals with ≤5 years of specialization was 34.2 ± 0.9 years and 53.0 ± 1.7 years for those with ≥15 years of specialization (Table 3).

**Table 3:** Comparison Between Years of Experience and Age.

Years of Experience	Mean	Median	Standard Deviation	CV	Min	Max	N	CI	P-value
≤5 years	34.2	33	4.0	12%	25	46	70	0.9	<0.001
6- 10 years	39.4	39	4.8	12%	33	65	49	1.4	
11-years	42.7	42	3.3	8%	38	53	40	1.0	
≥15 years	53.0	51	8.1	15%	42	72	88	1.7	

When comparing training time with demographic factors, there was significance between group coordination and the number of annual arthroscopies performed. Both had a higher number of annual arthroscopies and greater participation in training group coordination for those with ≥15 years of training (Table 4).

**Table 4:** Comparison Between Years of Experience and Distribution of Demographic Factors.

N		≤5 years		6- 10 years		11-14years		≥15 years		Total		P-value
		%	N	%	N	%	N	%	N	%	N	
Leads a training group	No	52	74.3%	31	63.3%	26	65.0%	45	51.1%	154	62.3%	0.028
	Yes	18	25.7%	18	36.7%	14	35.0%	43	48.9%	93	37.7%	
Holds TEOT – SBOT certification	No	0	0.0%	0	0.0%	0	0.0%	1	1.1%	1	0.4%	0.886
	Yes	70	100%	49	100%	40	100%	87	98.9%	246	99.6%	
Holds SBCOC certification	No	6	8.6%	2	4.1%	2	5.0%	4	4.5%	14	5.7%	0.713
	Yes	64	91.4%	47	95.9%	38	95.0%	84	95.5%	233	94.3%	
Sex	Female	1	1.4%	4	8.2%	2	5.0%	0	0.0%	7	2.8%	0.101
	Male	69	98.6%	45	91.8%	38	95.0%	88	100%	240	97.2%	
Number of shoulder arthroscopies performed per year	≤10	7	10.0%	3	6.1%	0	0.0%	2	2.3%	12	4.9%	<0.001
	11-20	14	20.0%	3	6.1%	4	10.0%	4	4.5%	25	10.1%	
	21-30	14	20.0%	10	20.4%	3	7.5%	2	2.3%	29	11.7%	
	31-50	13	18.6%	7	14.3%	6	15.0%	9	10.2%	35	14.2%	
	51-99	16	22.9%	15	30.6%	11	27.5%	32	36.4%	74	30.0%	
	≥100	6	8.6%	11	22.4%	16	40.0%	39	44.3%	72	29.1%	

According to the training method, the study did not present a statistically significant relationship with training time. There was consensus that the best (extremely valid) training methods are surgery performed as the lead surgeon, at 93.1%, followed by cadaver training at 81.4%. Courses, real simulators, and virtual simulators had the lowest frequency of “extremely valid” (Table 5).

**Table 5:** Comparison Between Years of Experience and Training Distribution.

N		≤5 years		6- 10 years		11-14years		≥15 years		Total		P-value
		%	N	%	N	%	N	%	N	%	N	
Surgery Performed as primary Surgeon	Neutral	0	0.0%	0	0.0%	0	0.0%	1	1.1%	1	0.4%	0.276
	Valid	2	2.9%	2	4.1%	2	5.0%	10	11.4%	16	6.5%	
	Extremely Valid	68	97.1%	47	95.9%	38	95.0%	77	87.5%	230	93.1%	
Cadaver surgery	Slightly Valid	0	0.0%	0	0.0%	0	0.0%	1	1.1%	1	0.4%	0.489
	Neutral	2	2.9%	0	0.0%	1	2.5%	4	4.5%	7	2.8%	
	Valid	13	18.6%	9	18.4%	2	5.0%	14	15.9%	38	15.4%	
	Extremely Valid	55	78.6%	40	81.6%	37	92.5%	69	78.4%	201	81.4%	
Observed surgery As Assistant	Not Valid	0	0.0%	0	0.0%	0	0.0%	1	1.1%	1	0.4%	0.121
	Slightly Valid	2	2.9%	0	0.0%	0	0.0%	1	1.1%	3	1.2%	
	Neutral	13	18.6%	2	4.1%	1	2.5%	7	8.0%	23	9.3%	
	Valid	24	34.3%	22	44.9%	13	32.5%	32	36.4%	91	36.8%	
	Extremely Valid	31	44.3%	25	51.0%	26	65.0%	47	53.4%	129	52.2%	
Courses	Not Valid	1	1.4%	0	0.0%	0	0.0%	2	2.3%	3	1.2%	0.792
	Slightly Valid	6	8.6%	2	4.1%	2	5.0%	6	6.8%	16	6.5%	
	Neutral	13	18.6%	6	12.2%	5	12.5%	18	20.5%	42	17.0%	
	Valid	29	41.4%	22	44.9%	22	55.0%	33	37.5%	106	42.9%	
	Extremely Valid	21	30.0%	19	38.8%	11	27.5%	29	33.0%	80	32.4%	
Literature	Not Valid	1	1.4%	1	2.0%	0	0.0%	5	5.7%	7	2.8%	0.594
	Slightly Valid	6	8.6%	4	8.2%	5	12.5%	4	4.5%	19	7.7%	
	Neutral	10	14.3%	9	18.4%	7	17.5%	10	11.4%	36	14.6%	
	Valid	22	31.4%	19	38.8%	15	37.5%	35	39.8%	91	36.8%	
	Extremely Valid	31	44.3%	16	32.7%	13	32.5%	34	38.6%	94	38.1%	
Physical simulators	Not Valid	0	0.0%	0	0.0%	0	0.0%	2	2.3%	2	0.8%	0.513
	Slightly Valid	3	4.3%	3	6.1%	4	10.0%	5	5.7%	15	6.1%	
	Neutral	8	11.4%	4	8.2%	1	2.5%	11	12.5%	24	9.7%	
	Valid	37	52.9%	21	42.9%	17	42.5%	36	40.9%	111	44.9%	
	Extremely Valid	22	31.4%	21	42.9%	18	45.0%	34	38.6%	95	38.5%	
Virtual simulators	Not Valid	0	0.0%	0	0.0%	0	0.0%	1	1.1%	1	0.4%	0.792
	Slightly Valid	6	8.6%	4	8.2%	3	7.5%	7	8.0%	20	8.1%	
	Neutral	16	22.9%	9	18.4%	5	12.5%	14	15.9%	44	17.8%	
	Valid	33	47.1%	20	40.8%	20	50.0%	35	39.8%	108	43.7%	
	Extremely Valid	14	20.0%	16	32.7%	12	30.0%	31	35.2%	73	29.6%	
Videos	Not Valid	1	1.4%	0	0.0%	0	0.0%	1	1.1%	2	0.8%	0.649
	Slightly Valid	1	1.4%	2	4.1%	4	10.0%	4	4.5%	11	4.5%	
	Neutral	8	11.4%	4	8.2%	1	2.5%	6	6.8%	19	7.7%	
	Valid	29	41.4%	21	42.9%	15	37.5%	42	47.7%	107	43.3%	
	Extremely Valid	31	44.3%	22	44.9%	20	50.0%	35	39.8%	108	43.7%	

In evaluating the number of arthroscopies by skill level, all agreed that for the SPECIALIST level, a total of over 500 arthroscopies is necessary, but for other levels, there was a difference between groups (Table 6).

**Table 6:** Comparison Between Years of Experience and Distribution of Shoulder Arthroscopy Volume by Skill Level.

N		≤5 years		6- 10 years		11-14years		≥15 years		Total		P-value
		%	N	%	N	%	N	%	N	%		
Specialist	6-10	0	0.0%	1	2.0%	0	0.0%	0	0.0%	1	0.4%	0.575
	11-20	0	0.0%	1	2.0%	0	0.0%	0	0.0%	1	0.4%	
	21-30	1	1.4%	1	2.0%	0	0.0%	2	2.3%	4	1.6%	
	31-50	0	0.0%	1	2.0%	0	0.0%	2	2.3%	3	1.2%	
	51-100	4	5.7%	2	4.1%	1	2.5%	6	6.8%	13	5.3%	
	101-500	24	34.3%	14	28.6%	8	20.0%	26	29.5%	72	29.1%	
	>500	41	58.6%	29	59.2%	31	77.5%	52	59.1%	153	61.9%	
Proficient	6-10	0	0.0%	1	2.0%	0	0.0%	0	0.0%	1	0.4%	0.026
	11-20	2	2.9%	1	2.0%	1	2.5%	1	1.1%	5	2.0%	
	21-30	2	2.9%	1	2.0%	0	0.0%	1	1.1%	4	1.6%	
	31-50	3	4.3%	1	2.0%	1	2.5%	2	2.3%	7	2.8%	
	51-100	28	40.0%	14	28.6%	2	5.0%	21	23.9%	65	26.3%	
	101-500	28	40.0%	25	51.0%	23	57.5%	39	44.3%	115	46.6%	
	>500	7	10.0%	6	12.2%	13	32.5%	24	27.3%	50	20.2%	
Competent	6-10	3	4.3%	1	2.0%	1	2.5%	1	1.1%	6	2.4%	<0.001
	11-20	3	4.3%	0	0.0%	0	0.0%	3	3.4%	6	2.4%	
	20-30	1	1.4%	0	0.0%	0	0.0%	0	0.0%	1	0.4%	
	21-30	11	15.7%	5	10.2%	1	2.5%	1	1.1%	18	7.3%	
	31-50	26	37.1%	13	26.5%	9	22.5%	22	25.0%	70	28.3%	
	51-100	17	24.3%	20	40.8%	7	17.5%	32	36.4%	76	30.8%	
	101-500	8	11.4%	9	18.4%	18	45.0%	27	30.7%	62	25.1%	
	>500	1	1.4%	1	2.0%	4	10.0%	2	2.3%	8	3.2%	
Secure	≤ 5	5	7.1%	1	2.0%	1	2.5%	1	1.1%	8	3.2%	0.001
	6-10	6	8.6%	2	4.1%	1	2.5%	4	4.5%	13	5.3%	
	11-20	16	22.9%	7	14.3%	6	15.0%	7	8.0%	36	14.6%	
	21-30	23	32.9%	9	18.4%	4	10.0%	22	25.0%	58	23.5%	
	31-50	11	15.7%	20	40.8%	8	20.0%	21	23.9%	60	24.3%	
	51-100	7	10.0%	9	18.4%	14	35.0%	25	28.4%	55	22.3%	
	101-500	2	2.9%	1	2.0%	5	12.5%	8	9.1%	16	6.5%	
	>500	0	0.0%	0	0.0%	1	2.5%	0	0.0%	1	0.4%	

## DISCUSSION

Our study demonstrated a number of 31 to 50 arthroscopies to perform a procedure SAFELY. Similar studies conducted in hip arthroscopies showed that the first significant reduction in both surgical time and complications can be observed after 30 cases of arthroscopy. Moreover, it was reported that patient outcomes and lower complication rates continued to improve at approximately 100 cases.^8-10^

Guttman et al.^11^evaluated the learning curve among shoulder surgeons in blocks of 10 rotator cuff repair surgeries and noted significant progress after the first block. Lim et al.^12^ demonstrated a significant decrease in rotator cuff re-tear rates according to the surgeon’s experience. Garstmans et al.^13^ also affirmed that the learning curve is a determining factor in surgical outcomes. In our study, we did not evaluate operative outcomes such as re-tear rates or clinical assessment of patients, but we did note an increase in surgery volume according to the surgeon’s experience, and once the surgeon has a higher total volume of procedures, they become more skilled with lower complication rates and better outcomes.

The complexity of an arthroscopic procedure varies according to the degree of injury and pathology involved. The surgical time required to repair extensive and chronic rotator cuff injuries is generally longer than for small to medium^14,15^ injuries, and as surgical experience is acquired, learning can be demonstrated by a quantitative decrease in operative time.^16^ Many authors use the time spent on the procedure as an evaluation element, but shorter surgical time is not necessarily related to better outcomes.^13^ In our study, we did not include surgical time or address any specific pathology to avoid many biases that similar studies have shown.^16-20^

McColl et al.^21^ showed that technological advancements had an impact on time and management during shoulder arthroscopy procedures; thus, we can consider that critical thinking regarding shoulder surgery may differ among surgeons of different generations. In our study, we did not observe significant differences in teaching tools among different generations of surgeons.

When evaluating participants’ viewpoints on the most valuable educational tools, respondents rated the lead surgeon role and cadaver training as the best techniques for acquiring skills. Similar findings have also been reported in several studies involving orthopedic surgeons and other arthroscopic procedures.^15-18^ Surprisingly, virtual simulators were rated as low-value tools for learning shoulder arthroscopy by respondents. Although these simulators have shown to improve overall arthroscopic performance in orthopedic residents, there are some technical limitations for certain real and virtual shoulder simulators. This is certainly an area that needs further exploration and study.

Technology can also produce simulators increasingly similar to the real procedure; however, we do not yet have any that reproduce the arthroscopic procedure attractively among orthopedic surgeons. Perhaps in the coming years, with technological advances, the perception of this type of tool may change.

The duration of the shoulder surgery specialization program in Brazil in services registered by the SBCOC is on average 12 months. However, this period is often insufficient for adequate training in arthroscopy. We know that learning depends on personal skills, but the provided structure has a significant influence on the learning process.

Inadequate training will produce an insufficient surgeon with a higher risk of complications, unsatisfactory results, and low productivity. The main limitation of the study was the number of participants included. Unfortunately, the adherence to the proposed questionnaire was very low, with only 39% of individuals who had correctly registered emails in the database responding to the questionnaire; the reason for non-adherence is unknown. No campaign was conducted through the website and social media to promote the research, which might have increased participation if the authors had used these tools. Additionally, the high number of incorrect email addresses also contributed to a lower number of participants. The authors of this study have already communicated with the SBCOC coordination to rectify the addresses to improve the society’s communication network.

Possibly the total number of orthopedic surgeons performing shoulder arthroscopy is underestimated when using the SBCOC database. Many surgeons perform this procedure and have never been registered with the society because they were trained in locations outside the society’s registration network.

We hope this work serves as a framework for the clinical validation of the shoulder arthroscopy learning curve among Brazilian surgeons and allows for the optimization of training methodologies.

## CONCLUSION

The study showed that most shoulder and elbow specialists in Brazil believe that 31 to 50 cases are necessary to perform the procedure safely, and over 500 cases to reach the specialist level. Participation as the lead surgeon and cadaver training were rated as very important in specialist training.

## Appendix 1.

I) Medical Registration

a) Email:

b) Sex: Male ( ) Female ( )

c) Age: ______ years

d) Region of practice in Brazil: South ( ) Southeast ( ) Central-West ( ) Northeast ( ) North( )

e) Do you coordinate or participate in any shoulder and elbow surgery training group (SBCOC)?

Yes ( ) No ( )

f) Years since completion of shoulder surgery training:

( ) Fellowship (2022)

( ) ≤5 years

( ) 6-10 years

( ) 11-14 years

( ) ≥15 years

g) Do you hold a TEOT certification?

Yes ( ) No ( )

h) What is the average number of shoulder arthroscopies you perform per year? (AS PRIMARY SURGEON)

( ) ≤10

( ) 11-20

( ) 21-30

( ) 31-50

( ) 51-99

( ) ≥100

II) Proficiency / Capability

a) What is YOUR level of skill in shoulder arthroscopy?

( ) Beginner: Very limited skill. Would have difficulty establishing portals

( ) Secure: oes not cause significant damage to cartilage, soft tissues or neurovascular structures. Would likely struggle to complete basic arthroscopic procedures.

( ) Competent: Able to reliably complete basic arthroscopic procedures, though may still exhibit inefficiencies in movement and execution.

( ) Proficient: Able to consistently and reliably complete both basic and more complex arthroscopic procedures.

( ) Expert: A distinguished leader in the field, recognized by peers for significant contributions to the advancement of surgical techniques.

b) How many shoulder arthroscopies do you believe must be PERFORMED to reach the SECURE level?

( ) ≤5

( ) 6-10

( ) 11-20

( ) 21-30

( ) 31-50

( ) 51-100

( ) 101-500

( ) >500

c) How many shoulder arthroscopies do you believe must be PERFORMED to reach the COMPETENT level?

( ) ≤5

( ) 6-10

( ) 11-20

( ) 21-30

( ) 31-50

( ) 51-100

( ) 101-500

( ) >500

d) How many shoulder arthroscopies do you believe must be PERFORMED to reach the PROFICIENT level?

( ) ≤5

( ) 6-10

( ) 11-20

( ) 21-30

( ) 31-50

( ) 51-100

( ) 101-500

( ) >500

e) How many shoulder arthroscopies do you believe must be PERFORMED to reach the EXPERT level?

( ) ≤5

( ) 6-10

( ) 11-20

( ) 21-30

( ) 31-50

( ) 51-100

( ) 101-500

( ) >500

III) Regarding shoulder arthroscopy training methods, what is your opinion?

Please select one: 1- not valid 2- slightly valid 3- neutral 4-valid 5- extremely valid

Through LITERATURE (reading articles, books, journals)

1- not valid

2- slightly valid

3- neutral

4-valid

5-extremely valid

Through VIDEO / Online Video / Video Lecture / Live Surgery

1- not valid

2- slightly valid

3- neutral

4-valid

5-extremely valid

Through FORMAL COURSES / ONLINE COURSES / CONFERENCES

1- not valid

2- slightly valid

3- neutral

4-valid

5-extremely valid

Through REAL SURGICAL SIMULATORS

1- not valid

2- slightly valid

3- neutral

4-valid

5-extremely valid

Through VIRTUAL SURGICAL SIMULATORS

1- not valid

2- slightly valid

3- neutral

4-valid

5-extremely

Through CADAVER SURGER

1- not valid

2- slightly valid

3- neutral

4-valid

5-extremely valid

Through SURGERY OBSERVED AS ASSISTANT

1- not valid

2- slightly valid

3- neutral

4-valid

5-extremely valid

Through SURGERY PERFORMED AS PRIMARY SURGEON

1- not valid

2- slightly valid

3- neutral

4-valid

5-extremely valid
